# Large transverse Hall-like signal in topological Dirac semimetal Cd_3_As_2_

**DOI:** 10.1038/srep27487

**Published:** 2016-06-06

**Authors:** Shih-Ting Guo, R. Sankar, Yung-Yu Chien, Tay-Rong Chang, Horng-Tay Jeng, Guang-Yu Guo, F. C. Chou, Wei-Li Lee

**Affiliations:** 1 Institute of Physics, Academia Sinica, Nankang, Taipei 11529, Taiwan; 2 Center for Condensed Matter Sciences, National Taiwan University, Taipei 10617, Taiwan; 3 Department of Physics, National Tsing Hua University, Hsinchu 30013, Taiwan; 4 Department of Physics, National Taiwan University, Taipei 10617, Taiwan

## Abstract

Cadmium arsenide (Cd_3_As_2_) is known for its inverted band structure and ultra-high electron mobility. It has been theoretically predicted and also confirmed by ARPES experiments to exhibit a 3D Dirac semimetal phase containing degenerate Weyl nodes. From magneto-transport measurements in high quality single crystals of Cd_3_As_2_, a small effective mass m^*^ ≈ 0.05 *m*_*e*_ is determined from the Shubnikov-de Haas (SdH) oscillations. In certain field orientations, we find a splitting of the SdH oscillation frequency in the FFT spectrum suggesting a possible lifting of the double degeneracy in accord with the helical spin texture at outer and inner Fermi surfaces with opposite chirality predicted by our *ab initio* calculations. Strikingly, a large antisymmetric magnetoresistance with respect to the applied magnetic fields is uncovered over a wide temperature range in needle crystal of Cd_3_As_2_ with its long axis along [112] crystal direction. It reveals a possible contribution of intrinsic anomalous velocity term in the transport equation resulting from a unique 3D Rashba-like spin splitted bands that can be obtained from band calculations with the inclusion of Cd antisite defects.

Topological materials have attracted great attention recently in condensed matter physics and material science. Non-trivial topology in a bulk band along with certain crystal symmetry can give rise to a novel material phase with unusual surface states, such as topological insulator^1^,^2^, topological crystalline insulator^3^,^4^ and Weyl semimetal (WS)^5^,^6^,^7^. Recently, a 3D Dirac semimetal (DS) phase has been theoretically predicted to exist in Na_3_Bi^8^, BiO_2_^9^, and Cd_3_As_2_^10^, where angle-resolved photoemission spectroscopy (ARPES) has provided direct evidences for such a 3D Dirac semimetal band^11^,^12^. As opposed to a WS, a DS contains overlapping Weyl nodes with opposite chirality in momentum space and typically requires a special crystal symmetry to protect the nodes against the gap-opening. The breaking of either time reversal symmetry or inversion symmetry in a DS is, therefore, a route toward realizing a WS phase, where a special magneto-electric coupling effect^13^ due to the non-conserving chiral current between separated Weyl nodes can lead to novel transport phenomena^14^,^15^,^16^,^17^,^18^. Cd_3_As_2_ contains inverted bands with ultra-high electron mobility, which were reported decades ago^19^,^20^, but only recently it revives as an example of 3D topological Dirac semimetals. It has been shown in experiments that a WS phase can be realized in Cd_3_As_2_ by applying intense magnetic field along the [001] crystal direction^21^,^22^. However, when the magnetic field is tilted away from [001] direction, the four-fold rotational symmetry is broken giving rise to gapped Dirac nodes in Cd_3_As_2_^23^. More recently, an exotic superconducting phase was discovered in point contact measurements on the surface of Cd_3_As_2_, which was attributed to the possible tip-induced symmetry-lowering or density variation near the point contact region^24^,^25^. Those results all point to an important fact that the system’s symmetry plays a crucial role dictating the details of Dirac band structures in Cd_3_As_2_.

In this study, we performed magnetotransport measurements on needle crystals of pure Cd_3_As_2_ grown by chemical vapor transport^26^,^27^. Compared to flux growth single crystals^16^,^28^, the CVT needle crystals have lower residual resistivity ratio suggesting higher defect level, which enables us to investigate the influence of defects to the Dirac band structures in Cd_3_As_2_. By introducing the Cd antisite defects into the band calculations, both the inversion symmetry and rotational symmetry were broken giving rise to a unique 3D Rashba-like spin splitted bands in Cd_3_As_2_, which may provide a qualitative explanation to the observed splitting in SdH frequency at various field angles and also a huge antisymmetric magnetoresistance (MR) with respect to magnetic fields in Cd_3_As_2_. Nevertheless, a possible current-jetting effect^29^,^30^ due to disorder and inhomogeneous conductivity, which has been reported in several narrow band-gap semiconductors^31^,^32^, will be discussed and compared to our transport data in Cd_3_As_2_.

## Results and Discussions

**Figure 1:**
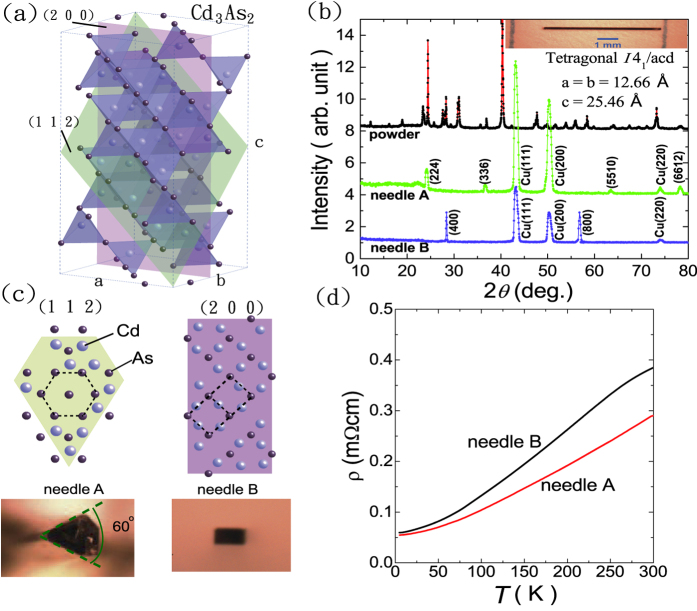
(**a**) An illustration of Cd_3_As_2_ crystal structure, where the (112) and (200) planes are shown. (**b**) Powder XRD pattern with preferred orientation fitted by I4_1_/acd space group symmetry. The single crystal XRD of needle A and B are also included to verify the corresponding needle long-axis directions to be along [112] and [200], respectively. The additional copper peaks come from the sample holder contribution. The upper inset shows the optical image of a 6 mm-long needle crystal of Cd_3_As_2_. (**c**) Optical images of needle A and B, showing triangular and rectangle cross-sections, where the corresponding (112) and (200) planes are illustrated in the upper panel. (**d**) Temperature dependence of the resistivity for needle A and needle B, showing similar metallic behavior.

The crystal structure of Cd_3_As_2_ as shown in Fig. 1(a) comprises a distorted antifluorite structure with ordered Cd vacancies, and it contains 80 atoms within a unit cell. For the phase stabilized at the lowest temperature, the Cd vacancies could be arranged orderly within a large tetragonal cell composed of corner-sharing CdAs_4_ tetrahedral units. The symmetry of Cd_3_As_2_ generated at the lowest temperature has been controversially indexed with either noncentrosymmetric space group I4_1_cd^33^ or I4_1_/acd with centrosymmetry^34^. While the indexing based on powder X-ray diffraction (XRD) is nearly equally satisfactory, and the initial Cd vacancy ordering is determinative on the final symmetry, the growth condition and crystal morphology could also play a role^34^. In the same batch of crystals, two types of needles can be identified. Needle A has nearly triangular-shape cross-section, while needle B has rectangle-shape cross-section as demonstrated in Fig. 1(c). Figure 1(b) shows the powder XRD pattern of the needle B crystal, which can be indexed with the space group I4_1_/acd with centrosymmetry. For needle A, the same space group of I4_1_/acd can be indexed in the X-ray diffraction. In order to identify the crystalline direction along the long axes of the needle crystals, a 4-circle diffractometer was used, and the corresponding single-crystal XRD of needle A and B are shown in Fig. 1(b), where the long axis directions of needle A and B were confirmed to be along [112] and [200], respectively. In addition, the full-width half maximum of the XRD peaks is merely about 0.2–0.4 degrees indicating good single crystalline quality with a small mosaic spread in our needle-like crystals. Crystallographically, it is reasonable to have stable phase of large (112) plane for Cd_3_As_2_, because (112) plane corresponds to a plane of pseudo-hexagonal close packing for a tetragonal unit cell with c ≈ 2a. The resistivity data shown in Fig. 1(d) indicate a metallic behavior with residual resistivity ratios (RRR ≡ *ρ*_300K_/*ρ*_5K_) of 4.4 and 5.6 for needle A and needle B, respectively. The corresponding electron density and the estimated Drude mobility are listed in Table 1. For needle B, the carrier density equals 9.2 × 10^17^ cm^−3^, and the corresponding Hall mobility *μ*_*D*_ is as high as 113,567 cm^2^V^−1^ s^−1^ at *T* = 5 K.

**Figure 2:**
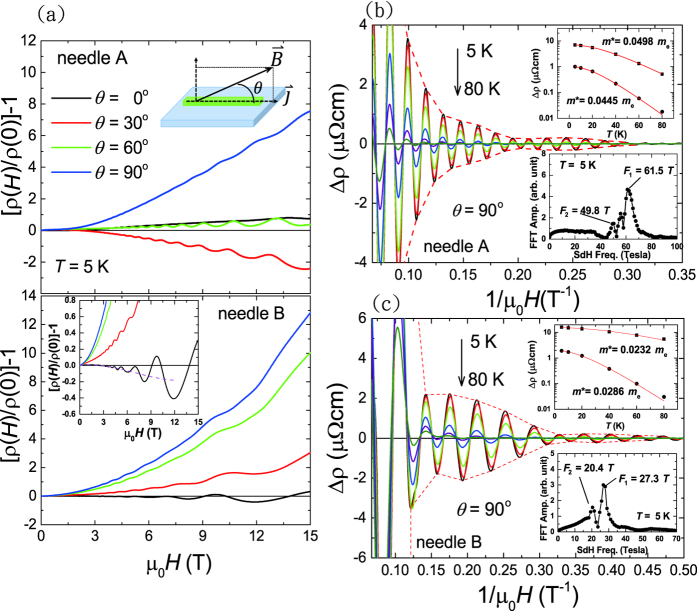
(**a**) Symmetrized MR in needle A (upper) and B (lower) at four different *θ* angles. The inset figure of lower panel is an enlarged view, showing a small negative MR at *θ* = 0°. (**b,c**) Plot the pure oscillatory component in MR versus 1/*μ*_0_*H* for needle A and B, respectively, at *θ* = 90° and various temperatures ranging from 5 to 80 K. The upper and lower insets are the corresponding effective masses fitting using Lifshitz-Kosevich formula and FFT spectra. Both needle A and B exhibit beating patterns in SdH oscillations with multiple closely-spaced peaks in the FFT spectra at *θ* = 90°.

Figure 2(a) shows the symmetrized MR (MR = [MR(*H*) + MR(−*H*)]/2) at four different *θ* values ranging from zero to 90 degrees, where *θ* is defined as the angle between the current and the applied magnetic field (upper inset cartoon). At *T* = 5 K, the magnitude of MR in needle B progressively decreases from a large positive value of MR ≡ [*ρ*(*H*)/*ρ*(0)] − 1 = 12.8 at *θ* = 90° to a small negative MR at *θ* = 0° angle (shown in the lower inset of Fig. 2(a)). On the contrary, the MR in needle A shows non-monotonic variation with *θ* values as demonstrated in the upper panel of Fig. 2(a). The unusual high *μ*_*H*_ enables the determination of band parameters via SdH oscillations in transport measurements. A large amplitude of SdH oscillation was found in MR at low *T*, which remains observable up to *T* ≥ 100 K. Figure 2(b) shows the pure oscillatory component of the resistivity Δ*ρ* versus 1/*μ*_0_*H* for needle A at 6 different temperatures ranging from 5 K to 80 K. The magnetic field is normal to the [112] direction. The damping of the SdH oscillation by temperature and magnetic field can be described by the following equation based on Lifshitz-Kosevich formula.


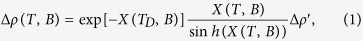


where *X*(*T*, *B*) ≡ 2*π*^2^*k*_*B*_Tm^*^/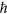
eB, m^*^ is the effective cyclotron mass and *T*_*D*_ is the Dingle temperature. Δ*ρ*′ refers to the undamped oscillatory component. By fitting the peak values of Δ*ρ*-*T* and log(Δ*ρ* ⋅ sin hX/X) − 1/*μ*_0_*H* according to Eq. 1, we determined m^*^ = 0.0498 *m*_*e*_ for needle A as demonstrated in the upper inset of Fig. 2(b). Two resolvable SdH frequencies of 49.8 and 61.5 Tesla were identified in the Fast Fourier transform (FFT) spectrum shown in the lower inset of Fig. 2(b). On the other hand, the pure oscillatory component of resistivity for needle B is shown in Fig. 2(c), where two SdH frequencies of 20.4 and 27.3 Tesla and m^*^ = 0.0232 *m*_*e*_ were determined. The calculated band for I4_1_/acd structure with Cd antisites in (112) plane at similar *n*_*e*_ gives two close SdH frequencies *F*_1_ and *F*_2_, which is in relatively good agreement with the experimental data. A summary of the band parameters for needle A and needle B from SdH experiments and calculation is given in Table 1.

**Figure 3:**
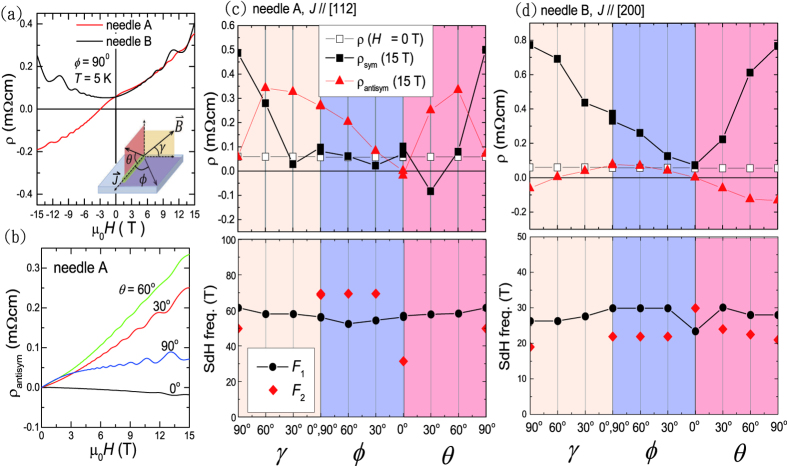
(**a**) Resistivity *ρ* versus field *μ*_0_*H* shows significant antisymmetric component in needle A and B. The lower inset cartoon illustrates the geometric definition for angles of *γ*, *ϕ*, and *θ*. (**b**) Extracted antisymmetric components (*ρ*_*antisym*_) in MR of needle A at four different *θ* angles. At *μ*_0_*H* = 15 Tesla, the angular dependence of extracted symmetric (*ρ*_*sym*_) and antisymmetric (*ρ*_*antisym*_) components of needle A and B are shown in (**c,d**), respectively. The lower panel shows the corresponding SdH frequencies that can be identified from the FFT spectra. The major SdH frequencies *F*_1_ (black circles) at about 61 Tesla and 27 Tesla for needle A and B, respectively, both show weak angular dependence. Secondary SdH frequencies *F*_2_ appear at some angles shown as red diamonds.

Figure 3(a) shows the resistivity versus magnetic field applied along *ϕ* = 90°, where the definition of corresponding angles of *γ*, *ϕ*, and *θ* are illustrated in the lower inset cartoon. Both needle A and needle B exhibit noticeable antisymmetric component in the MR regardless of the large aspect ratio (≡ length/width) of ≅3.9 (15.7) in needle A (B). By using the formula of *ρ*_*sym*_ ≡ [*ρ*(*H*) + *ρ*(−*H*)]/2 and *ρ*_*antisym*_ ≡ [*ρ*(*H*) − *ρ*(−*H*)]/2, symmetric (*ρ*_*sym*_) and antisymmetric (*ρ*_*antisym*_) components of the MR can be extracted. The resulting field dependence of *ρ*_*antisym*_ in needle A at four different *θ* angles is shown in Fig. 3(b), where the corresponding *ρ*_*sym*_ has been shown in Fig. 2(a). The upper panels of Fig. 3(c,d) are the angular dependence of the extracted *ρ*_*sym*_ and *ρ*_*antisym*_ at *μ*_0_*H* = 15 T for needle A and needle B, respectively. We remark that, for needle B with current along the [200] crystal direction, *ρ*_*antisym*_ at some angles can be even larger than *ρ*_*sym*_, and the magnitude of *ρ*_*antisym*_ appears to be at minimum when *ϕ* = 0° or *θ* = 0°. The SdH frequencies determined from the FFT spectra for needle A and needle B are shown in the lower panels of Fig. 3(c,d), respectively, where apparent multiple SdH frequencies can be identified. The major SdH frequencies *F*_1_ (black circles) being the location of largest peak in the FFT spectrum were determined to be about 61 Tesla and 27 Tesla in needle A and needle B, respectively, exhibiting weak dependency on *γ*, *ϕ*, and *θ* values. We also note that secondary SdH frequencies (red diamonds) can be clearly observed at some angles in both needle A and needle B.

**Figure 4:**
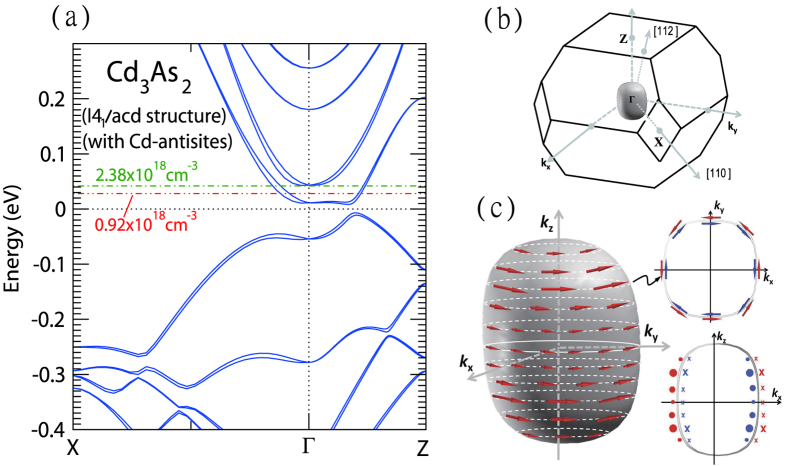
(**a**) A calculated band structure of Cd_3_As_2_ with I4_1_/acd space group symmetry and Cd antisite defects. The two dotted lines correspond to the Fermi level locations for needle A and B, respectively, determined from the experimental values of Fermi surface area. (**b**) The calculated 3D Fermi surface with an energy of ≈0.04 eV above the gapped Dirac node close to the Fermi level of our samples suggests a slightly distorted Fermi sphere in Cd_3_As_2_. [112] and [200] crystal directions are indicated corresponding to the long-axis directions for needle A and B, respectively. (**c**) The spin texture over an outer Fermi surface. Red and blue arrows indicate spin texture on the outer and inner Fermi surfaces, respectively, where the size of the arrows indicates the spin polarization. The lower inset shows the spin texture profile on *k*_*x*_ − *k*_*z*_ plane, while the upper inset is a top view of the Fermi surfaces at finite *k*_*z*_ indicated by the black arrows.

Figure 4(a) shows the calculated band structure based on I4_1_/acd space group symmetry with Cd antisite defects. The dotted-dash lines represent two Fermi level locations set by experimental SdH frequencies within the rigid band approximation. For needle B with a density of *n*_*e*_ = 0.92 × 10^18^ cm^−3^, the Fermi level is about 30 meV above the Dirac node. As described previously, the Cd vacancies are ordered in the manner to keep the inversion symmetry in the defect-free I4_1_/acd lattice. The inversion symmetry then leads to the spin degenerate Dirac bands, which can not explain the observed splitted SdH frequencies. Inspired by our calculated spin splitted Dirac bands of the noncentrosymmetric I4_1_/cd lattice (see supplementary information), we thus introduce ≈1% Cd antisite defect in the centrosymmetric I4_1_/acd lattice by moving one Cd ions to one of the Cd vacancies so as to break the inversion symmetry and split the centrosymmetry protected spin degenerate Dirac bands. As shown in the figure, a lifting of spin degeneracy in the calculated band in the *k*_*x*_ − *k*_*y*_ plane is successfully obtained from breaking of the inversion center of I4_1_/acd structure by the Cd antisite defects and thus gives rise to the two calculated SdH frequencies listed in Table 1. This is in accord with the observed beating patterns and multiple closely-spaced SdH frequencies shown in Fig. 2(b,c) (see also supplementary information). However, we note that a small gap about 10 meV is opened at the Dirac node due to the rotational symmetry breaking by the Cd antisites. In addition, the band width was reduced causing a flatter Dirac bands, which results in a somewhat lower Fermi level location in our band calculations compared to previous reports based on defect-free band calculations^12^,^28^. In the lower panels of Fig. 3(c,d), the variation of the SdH Frequency with *γ*, *θ*, and *ϕ* is less than 15%. The Fermi surface can, therefore, be regarded as a slightly distorted sphere as illustrated in Fig. 4(b) obtained from a calculated band with a Fermi level at ≈0.04 eV above the gapped Dirac node for the I4_1_/acd structure with ≈1% Cd antisite defects. The shape of the Fermi surfaces and the spin chirality are similar to those of the defect-free noncentrosymmetric I4_1_cd lattice at 0.1 eV with smaller band splittings. This remains well above the Lifshitz transition occurring at around 0.01 eV. Hence, the two Fermi pockets corresponding to two gapped Dirac nodes along the Γ − Z line merged together into a bigger Fermi surface centered at Γ. The [112] and [200] directions corresponding to the long axes of needle A and needle B, respectively, are also indicated in Fig. 4(b). We further performed calculation on spin texture of the 3D Fermi surface shown in Fig. 4(c), where the electrons over the Fermi surfaces possess chirality with a spin-momentum lock in-plane spin texture. Furthermore, due to the broken centrosymmetry by the Cd antisites, the spin texture on the inner and outer Fermi surfaces revolve in the opposite direction as shown in the upper inset of Fig. 4(c) similar to a Rashba-like band-splitting. The difference in the area of the two Fermi surfaces over the ab-plane is about 29%, which is compatible with the experimental value (≈20%) from splitted SdH frequencies shown in Fig. 2(b,c). We remark that such an unusual 3D Rashba-like spin splitted band is a direct consequence of the large spin-orbit coupling and also Cd-antisites induced inversion symmetry breaking in Cd_3_As_2_ while the time reversal symmetry remains valid.

The raw data of the resistivity shows an antisymmetric behavior with respect to the in-plane magnetic field inferring a large transverse Hall-like signal picked up by the voltage leads due to the leads misalignment. The estimation of normal Hall signal due to leads misalignment is difficult due to unknown distance *W*′ between leads normal to the current direction. A rough estimation of normal Hall contribution can be given using *ρ*_*xy*,*norm*_ = *ρ*_*xy*_ × (*W*′/*W*)/(*AR*), where W is the needle width and AR = L/W is the aspect ratio of the needle. If we take *W*′/W ≈ 10% and AR = 3.9 (15.7) for needle A(B), *ρ*_*xy*,*norm*_ (15 T) = 0.1 mΩcm (0.06 mΩcm), which is at least 4-fold smaller in magnitude compared to the observed *ρ*_*antisym*_ shown in Fig. 3(c,d). In addition, *ρ*_*xy*,*norm*_ should grow monotonically with increasing magnetic field strength normal to the current direction (i.e., the *θ* and *ϕ* angles go from zero to 90 degrees), which contradicts with the non-monotonic variation of *ρ*_*antisym*_ with *θ* values observed in needle A shown in Fig. 3(c).

Now we turn to discuss the possible current-jetting effect. It was shown that the inhomogeneous conductivity can cause a distortion in the current path giving rise to a perpendicular current component 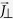 that flows normal to the major current direction 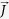
30,31. In Cd_3_As_2_, high resistivity anisotropy and relatively short quantum scattering lifetime of ~10^−14^ s as determined from SdH oscillations were reported^16^,^35^ making the current jetting effect a likely source of unusual magnetotransport behavior. In general, 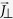 contributes a normal Hall signal to MR resulting in a *B*-linear and non-saturating MR in transverse field with 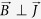. For longitudinal field geometry of 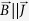, further bending in 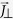 by 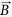 results in a decrease in potential drop between voltage electrodes and hence a negative MR. Compared to the MR data of Cd_3_As_2_ shown in Fig. 2(a), a nearly *B*-linear MR at *θ* = 90° (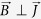) only appeared at fields higher than about 10 Tesla. In addition, a positive MR at *θ* = 0° (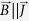) with vanishing *ρ*_*antisym*_ up to 15 Tesla (Fig. 3(b)) was observed in needle A instead. Those phenomena cannot be fully understood via the current-jetting mechanism alone. We also note the large negative MR found in needle A at *θ* = 30°, where its origin is unclear and requires further investigation. On the other hand, *ρ*_*antisym*_ remains finite at low-field diffusive limit and grows larger in higher fields without saturation as shown in Fig. 3(b). In particular, the *ρ*_*antisym*_ − *μ*_0_*H* curve at *θ* = 90° exhibits a slower increase of *ρ*_*antisym*_ at higher fields, which is different from others being nearly linear with field. Those findings strongly suggests that there is additional contribution to the antisymmetric MR other than the normal Hall contribution and the high field effect in quantum limit. We also find that such an antisymmetric behavior in MR persists up to room temperature and shows relatively weak *T* dependence (see supplementary information), which supports for a more intrinsic origin rather than due to impurity effect at low *T*.

Another likely mechanism is the anomalous velocity term associated with the non-zero Berry curvature of the bulk band^36^,^37^. In a general formalism, the electron velocity can be expressed as 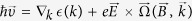, where 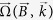 is the Berry curvature. In a diffusive transport^14^, it can be shown to give a total current of


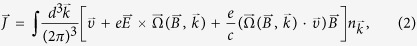


where 
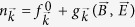
 is the electron distribution function. 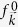 and 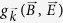 are the equilibrium and non-equilibrium distribution function, respectively. In zero field, time reversal symmetry ensures that the integration of anomalous velocity term (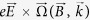) with 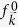 over the whole Fermi surface gives zero contribution to the total current. However, in the presence of magnetic field and electric bias, the induced 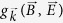 can result in non-vanishing anomalous velocity contribution to the total current as shown in Eq. 2. It is known that the electric current and spin polarization can couple with each other in a 2D electron gas system with Rashba spin splitting^38^,^39^. For example, current-induced spin polarization has been demonstrated providing a strong support for the observable effects originating from non-equilibrium 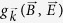 term. In the case of a 3D Rashba-like spin splitted band shown in Fig. 4(c), 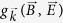 value can have nontrivial dependence on the orientation of 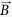 and 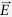, which results in anomalous angular dependence in the transport phenomena.

**Figure 5:**
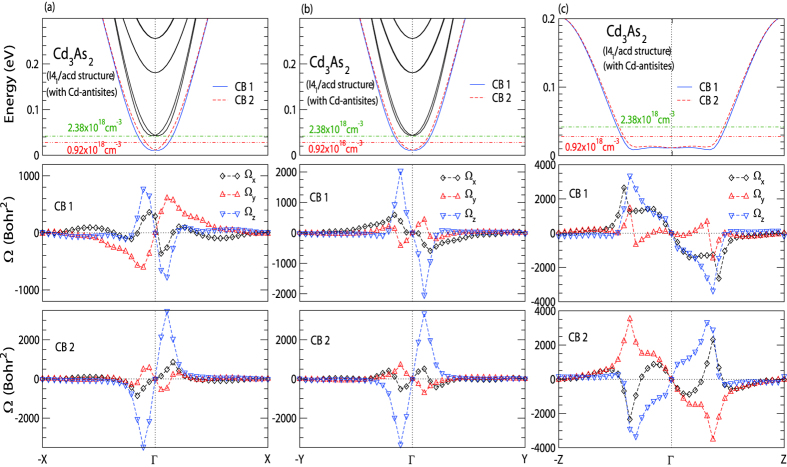
Berry curvature calculations along Γ − *X* (**a**), Γ − *Y* (**b**), and Γ − *Z* (**c**) using the band structure show in Fig. 4(a). The upper panels show the band structures. The corresponding Berry curvature 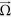 for CB 1 and CB 2 bands are shown in the middle and lower panels, respectively. Non-zero Berry curvatures were found along all three principle axes in accord with a 3D nature of the Rashba-like spin-splitted band in Cd_3_As_2_ with Cd antisites.

In order to look into the anomalous velocity contribution, Berry curvature 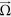 based on the band structure shown in Fig. 4(a) was calculated. Figure 5(a–c) shows the results along Γ − *X*, Γ − *Y*, and Γ − *Z*, respectively. We note that 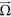 for the two conduction bands of CB1 (middle panel) and CB2 (lower panel) have non-zero magnitudes along all three principle axes indicating a 3D nature of the Rashba-like spin-splitted band. 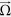 is antisymmetric with respect to the Γ point as expected for a system with time reversal symmetry. We remark that the calculated 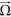 gives the largest non-zero value along Γ − *Z* direction, which is nearly 4-fold bigger compared to that along Γ − *X*. Such a difference can provide qualitative explanation on the observation of a larger *ρ*_*antisym*_ in needle A sample with current applied along [112], where a non-zero electric bias along Γ − *Z* is present. We note that similar splitting in SdH frequency was also reported in flux-growth Cd_3_As_2_ crystals^28^. It was attributed to the two nested ellipsoidal Fermi surface along the Γ − *Z*, showing single SdH frequency when the magnetic field is along [112] direction, which is not compatible with our observation in needle A that shows two distinct SdH frequencies at *θ* = 0°. It is also not clear how the spin-degenerate and nested ellipsoidal Fermi surface can lead to a large transverse current response in the charge transport.

In conclusion, Cd_3_As_2_ exhibits a 3D topological Dirac semimetal phase with ultra-high electron mobility. According to our band calculations, Cd antisite defects can be an effective symmetry-breaking mechanism giving rise to a unique 3D Rashba-like spin splitting in the *k*_*x*_ − *k*_*y*_ plane. This is also supported by our angular SdH measurements, showing splitted Fermi surface. By comparing the transport data in needle A and B with long axes along [112] and [200], respectively, we uncover significant transverse Hall-like signals in MR, which can not be simply attributed to the normal Hall-effect-related mechanisms. Particularly, we found that such a transverse Hall-like signal is much more pronounced in needle A with current bias along [112] direction. This can be qualitatively understood from our calculated Berry curvature, showing a 4-fold enhancement in magnitude along Γ − *Z*, and the corresponding non-equilibrium electron distribution due to the external magnetic field and electric bias can lead to an unusual large anomalous velocity contribution to the electron transport.

## Additional Information

**How to cite this article**: Guo, S.-T. *et al*. Large transverse Hall-like signal in topological Dirac semimetal Cd_3_As_2_. *Sci. Rep*. **6**, 27487; doi: 10.1038/srep27487 (2016).

## Supplementary Material

Supplementary Information

## Figures and Tables

**Table 1:** Major SdH frequency *F*_1_(T), secondary SdH frequency *F*_2_(T), effective mass m^*^(*m*_*e*_), and Drude mobility *μ*_*D*_ (cm^2^V^−1^ s^−1^) obtained from SdH oscillation measurements and calculated band structure.

	*n*_*e*_ × 10^18^ (cm^−3^)	F_1_ (T)	F_2_ (T)	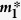 (*m*_*e*_)	*μ*_*D*_ at 5 K (cm^2^V^−1^ s^−1^)
needle A	2.4	61.5	49.8	0.0498	45,577
needle B	0.92	27.3	20.4	0.0232	113,567
Calculation	2.4	52	35	–	–
	0.92	31	18	–	–
