# Supplementary material for: Large transverse Hall-like signal in topological Dirac semimetal Cd3As2
Source: Sci Rep. 2016 Jun 6;6:27487. doi: 10.1038/srep27487 (PMC4893742; doi:10.1038/srep27487)
Supplement: Supplementary Information [file srep27487-s1.pdf]

Supplementary Information

Large transverse Hall-like signal in topological Dirac semimetal Cd_3As_2

Shih-Ting Guo, R. Sankar, Yung-Yu Chien, Tay-Rong Chang, Horng-Tay Jeng, Guang-Yu Guo, F. C. Chou, and Wei-Li Lee

1. Single crystal Cd_3As_2 **growth.** Single crystal Cd_3As_2 has been grown with chemical vapor transport method. Mixture of cadmium and arsenic elements of 6N purity in weight ratio of Cd(69%): As(31%) were sealed in an evacuated quartz tubing after several times of argon gas purging. Preliminary melting and homogenizing was done at 800°C with a slow heating rate of 3 °C/min and then furnace cooled to room temperature. Vapor transport procedure was performed using sealed quartz tubes (ID=1.8 cm and 30 cm long) in a horizontal tube furnace with preset temperature gradient. The optimum temperature profile for the growth of Cd_3As_2 through vapor phase was found to be a three-zone temperature setting of T_1 =600°C , T_2=570°C , and T_3=500°C with an average gradient of 1.7 °C/cm between 500-600°C. Single crystals can be collected near the T_3 zone center. The growth rate was estimated to be approximately 0.5 g/day for total amount of precursor near 70 grams. Similar growth for the Sn and Zn substituted crystals followed the same recipe using precursors of stoichiometric molar ratios.

2. Band structure calculations. *Ab initio* relativistic band structure calculations based on the generalized gradient approximation [1] were carried to investigate the fermiology as well as the spin texture over the Fermi surface. The experimental $I4_1cd$ and $I4_1/acd$ crystal structures were adopted and the accurate full-potential projector-augmented wave method, as implemented in the VASP package [2, 3] was used. The self-consistent calculations with the energy cutoff of about 300 eV were performed using a 8×8×8 Monkhorst-Pack k-mesh.

3. The beating patterns in SdH oscillation

In order to avoid possible artifact in subtracting the non-oscillartory component of resistivity ρ, we start with the raw data of ρ versus $1/\mu_0 H$. The oscillating component can be seen more clearly by taking the first derivative of ρ with respect to $1/\mu_0$, which effectively removes the non-oscillatory contribution that is linear in $1/\mu_0 H$. The resulting $d\rho/d(1/\mu_0 H)$ versus $1/\mu_0 H$ curve is then used for the FFT analysis. Figure S1(a) and (b) are the results for needle A using the method described above, where the magnetic field is applied along the in-plane direction at different ϕ values. In Fig. S1(a), apparent beating patterns were

observed at $\phi = 90^o$, 75°, 60° and 0°. The corresponding FFT spectra shown in Fig. S1(b) give two peaks in all ϕ angles. The curves at different ϕs were vertically shifted, and the FFT spectrum for $\phi = 0^o$ was amplified by 3 times for clarity. The arrows in Fig. S1(a) indicate the apparent node location for the beating pattern in SdH oscillations.

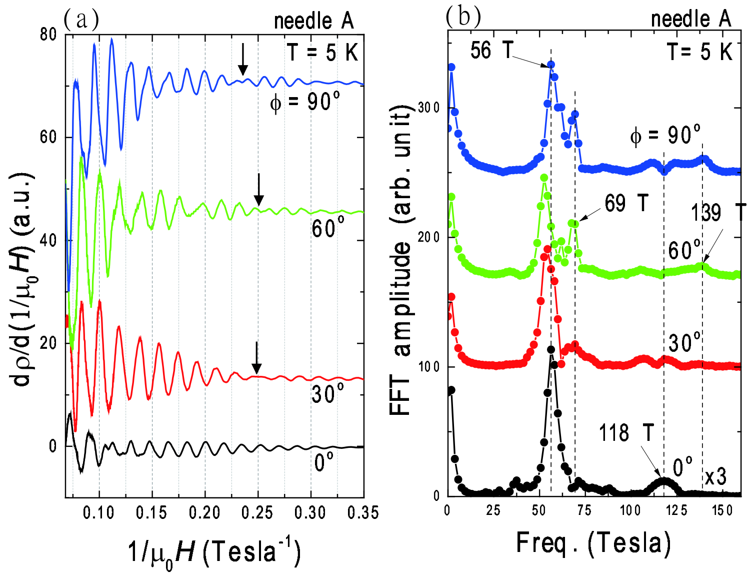

FIG. S1: (color online) (a) $d\rho/d(1/\mu_0 H)$ versus $1/\mu_0 H$ curve for needle A at different ϕs and T = 5 K. The arrows indicate the apparent node location for the beating pattern in SdH oscillations. (b) shows the corresponding FFT spectra of the SdH oscillation in (a).

4. Comparison of the calculated band structures for the $I4_1/acd$ and $I4_1cd$ structures.

The two most probable space groups of Cd_3As_2 found to date are $I4_1cd$ (No.110) and $I4_1/acd$ (No.142). These two symmetries are difficult to distinguish when assigning space group based on systematic absence of diffraction peaks alone. The key difference between them is that $I4_1/acd$ exhibits the centro-symmetry, whereas $I4_1cd$ does not. The lack of centro-symmetry in $I4_1cd$ structure, however, yields profound influence on the band structure. As shown in the left panel in Fig. S2, the degeneracies of the Dirac bands in the k_x-k_y plane are lifted into spin up (blue circles) and spin down (red circles) bands by the broken centro-symmetry in $I4_1cd$ space group. Although the splittings in energy are small ($\approx$ 5 meV), the spin components are clearly separated, resulting in two spin polarized Fermi surfaces with slightly different sizes. The difference in the area of the Fermi surfaces at $\approx$ 0.1 eV above the Dirac node projected onto the a-b plane is about 5 %. On the other hand,

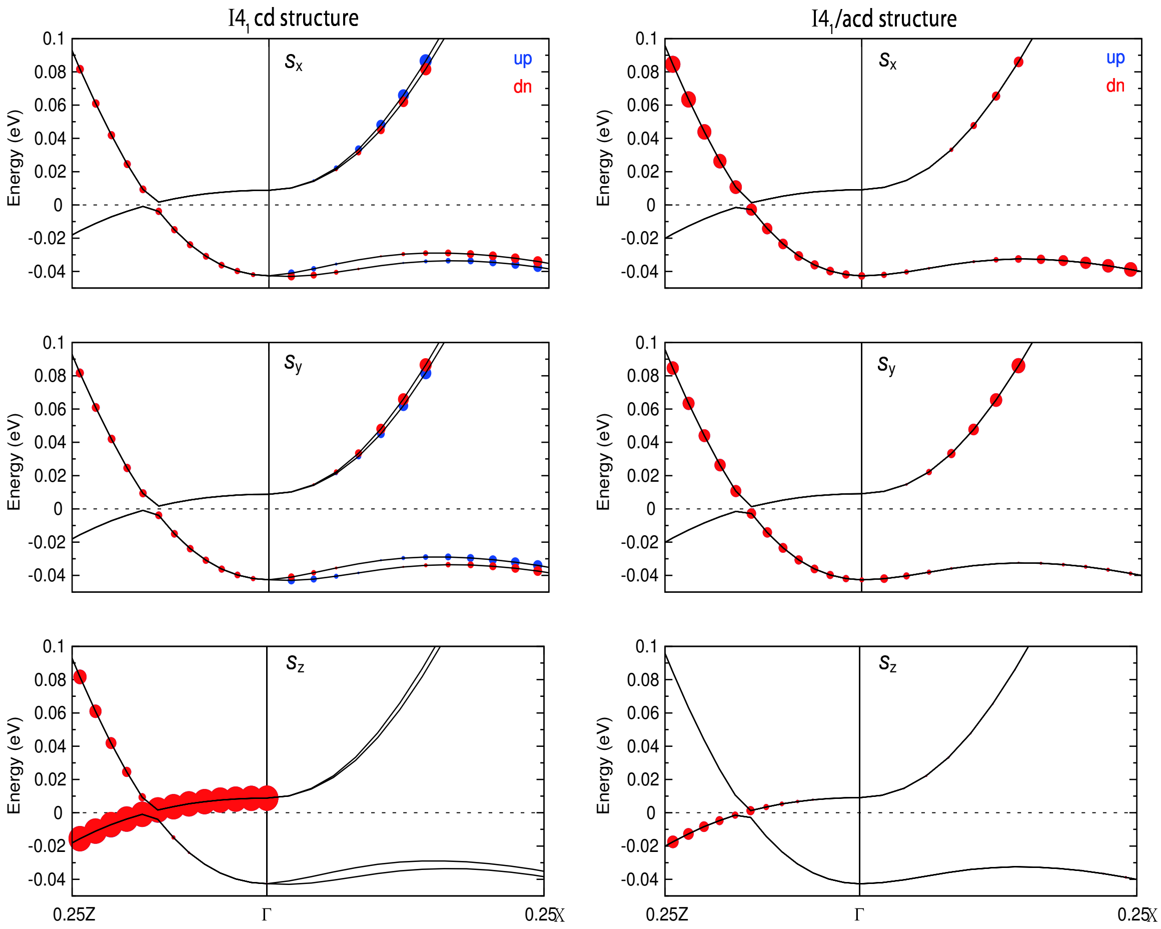

FIG. S2: (color online) Band structures projected onto the S$_x$, S$_y$, and S$_z$ spin components along Z-Γ-X high symmetry lines in the Brillouin zone for I4$_1$cd (left panel) and I4$_1$/acd (right panel) symmetries. The size of the blue and red circles denote the magnitude of spin up and spin down components.

because of the centro-symmetry in the I4$_1$/acd lattice, the Dirac bands and the spin states are perfectly degenerate (the blue circles are perfectly covered by the red ones) no mater along ΓX or ΓZ as shown in the right panel of Fig. S2. Hence only one spin degenerate Fermi surface can be detected. Nevertheless, we remark that spin-splitted bands can also appear in I4$_1$/acd by taking into account the Cd antisite defects that break the centro-symmetry as shown in Fig. 4(a) of the main text, which turns out to give larger spin-splitted bands compared to pure I4$_1$cd. This is presumably the origin of the observed double-peak in the FFT spectrum of our SdH oscillation measurements.

5. The field and temperature dependencies of $\rho_{antisym}$

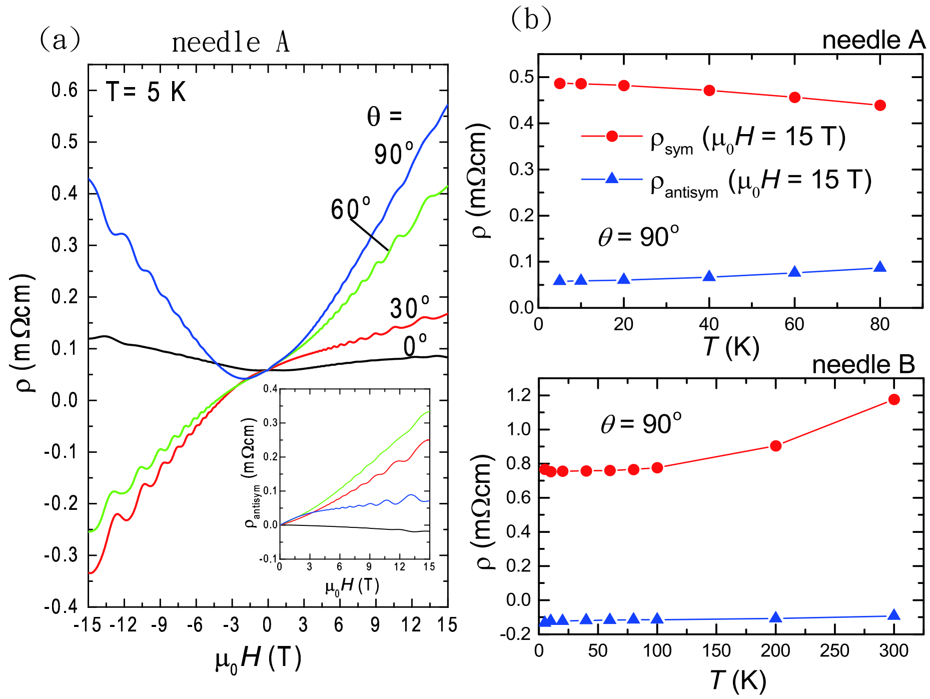

FIG. S3: (color online) Resistivity as a function of magnetic field at T = 5 K in needle A with different θs ranging from 0° to 90°. (b) The T dependence of the ρ_{sym} (circle) and $\rho_{antisym}$ (triangle) at $\theta = 90°$ in needle A (upper panel) and B (lower panel).

Figure S3(a) shows the raw data of the resistivity versus magnetic field at 4 different θ angles ranging from 0 degree to 90 degree. We remark that the ρ-$\mu_0 H$ curves exhibit large antisymmetric component with respect to $\mu_0 H$ at $\theta = 60°$ and 30°, which is also true in the weak field regime with $\mu_0 H \leq 1$ T. By taking the antisymmetric component of the raw data, the resulting $\rho_{antisym}$ versus $\mu_0 H$ is shown in the lower inset of Fig. S3(a). For θ = 60° and 30°, $\rho_{antisym}$ grows larger at higher field. On the other hand, $\rho_{antisym}$ tends to saturate for $\mu_0 H \geq 3$ T at $\theta = 90°$. The non-linear field dependence of $\rho_{antisym}$ and also the non-monotonic variation of $\rho_{antisym}$ with increasing θ values suggest a non-trivial origin of $\rho_{antisym}$ that can not be solely explained by a single band Hall effect contribution. We also studied the temperature dependence of $\rho_{antisym}$. As demonstrated in Fig. S3(b), the antisymmetric component of MR ($\rho_{antisym}$) in both needle A (upper panel) and B (lower panel) show relatively weak T dependence compared to the symmetric component (ρ_{sym}). This may suggest an intrinsic origin for the large $\rho_{antisym}$ observed in Cd_3As_2, and the (magnetic) impurity effect, which can be significant at low T, does not play an important role.

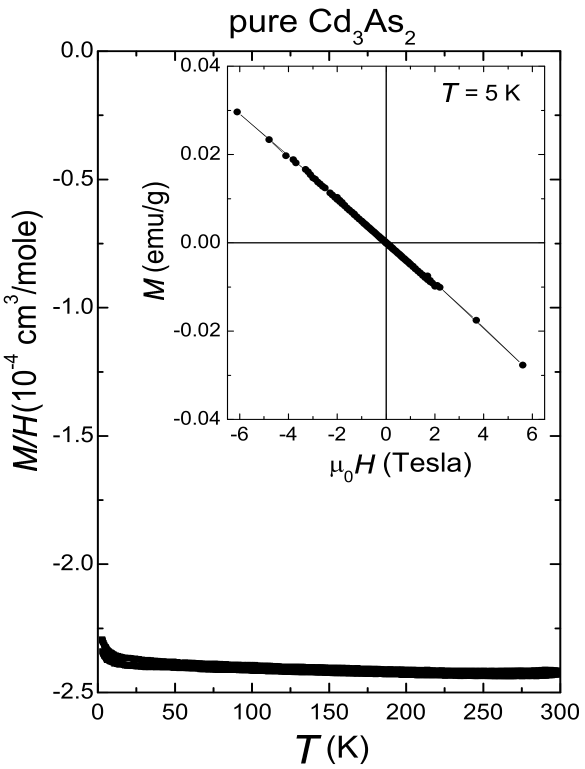

FIG. S4: (color online) Average magnetic susceptibility $\chi = M/H$ versus temperature of pure Cd_3As_2 with magnetic field of 1000 Oe applied parallel to the (112)-plane. The inset shows the magnetization isotherm $M(T)$ at T = 5K.

6. The average spin susceptibility of pure Cd_3As_2

The average spin susceptibility (M/H) of pure Cd_3As_2 measured by a SQUID magnetometer is shown in Fig. S4. It exhibits a nearly temperature-independent diamagnetic response at the level of -2.4 $\times$ 10^{-4} cm^3/mole. The small increase below $\approx$ 10 K could be due to the paramagnetic contribution from isolated spins of minor impurities and defects. The inset of Fig. S4 shows the magnetization isotherm (M(H)) at T = 5 K, which clearly shows a diamagnetic behavior up to 6 Tesla without hysteresis. Since the core diamagnetic contribution of Cd_3As_2 is estimated to be -7.8$\times$$10^{-5}$ cm^3/mole, and usually the Van Vleck paramagnetic contribution is of the same order, the observed large diamagnetic susceptibility is uncommon. Unusually high diamagnetism has been found in some of the hybridization gap materials, but the fundamental mechanism remains to be clarified [4].

7. Measurement geometry for needle crystals of Cd_3As_2

The transport measurements were performed by standard four-probe technique. Fig.

S5 shows the optical images for needle A (upper panel) and needle B (lower panel) with contact electrodes. The corresponding cross-sectional views are shown on the right images. The distance L between the voltage leads, the width W and the height h of the needle crystals are also shown in the figure. The aspect ratios AR $\equiv$ L/W for needle A and B are 3.9 and 15.7, respectively.

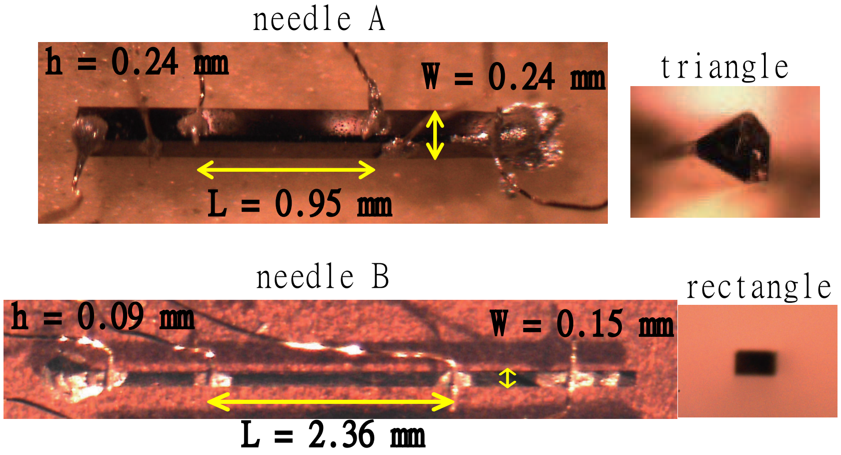

FIG. S5: (color online) Optical images of needle crystals with contact electrodes. The right images show the corresponding cross-sectional shapes of the needle crystals.

[1] Perdew, J.P., Burke, K., & Ernzerhof, M. *Phys. Rev. Lett.* **77**, 3865 (1996).

[2] Kresse, G. & Hafner, J. *Phys. Rev. B* **48**, 13115 (1993).

[3] Kresse, G. & Furthmüller, J. *Comput. Mater. Sci.* **6**, 15 (1996).

[4] Mandrus, D., Keppens, V., Sales, B., & Sarrao, J. *Phys. Rev. B* **58**, 3712 (1998).
